# Residual networks models detection of atrial septal defect from chest radiographs

**DOI:** 10.1007/s11547-023-01744-0

**Published:** 2023-12-11

**Authors:** Gang Luo, Zhixin Li, Wen Ge, Zhixian Ji, Sibo Qiao, Silin Pan

**Affiliations:** 1 Heart Center, Women and Children’s Hospital, Qingdao University, 6, Tongfu Road, Qingdao, 266034 China; 2 Department of Radiology, Women and Children’s Hospital, Qingdao University, Qingdao, 266034 China; 3 The School of Computer Science and Technology, China University of Petroleum, Qingdao, 266580 China

**Keywords:** Residual networks, Atrial septal defect, Digital radiography, Chest

## Abstract

**Object:**

The purpose of this study was to explore a machine learning-based residual networks (ResNets) model to detect atrial septal defect (ASD) on chest radiographs.

**Methods:**

This retrospective study included chest radiographs consecutively collected at our hospital from June 2017 to May 2022. Qualified chest radiographs were obtained from patients who had finished echocardiography. These chest radiographs were labeled as positive or negative for ASD based on the echocardiographic reports and were divided into training, validation, and test dataset. Six ResNets models were employed to examine and compare by using the training dataset and was tuned using the validation dataset. The area under the curve, recall, precision and F1-score were taken as the evaluation metrics for classification result in the test dataset. Visualizing regions of interest for the ResNets models using heat maps.

**Results:**

This study included a total of 2105 chest radiographs of children with ASD (mean age 4.14 ± 2.73 years, 54% male), patients were randomly assigned to training, validation, and test dataset with an 8:1:1 ratio. Healthy children’s images were supplemented to three datasets in a 1:1 ratio with ASD patients. Following the training, ResNet-10t and ResNet-18D have a better estimation performance, with precision, recall, accuracy, F1-score, and the area under the curve being (0.92, 0.93), (0.91, 0.91), (0.90, 0.90), (0.91, 0.91) and (0.97, 0.96), respectively. Compared to ResNet-18D, ResNet-10t was more focused on the distribution of the heat map of the interest region for most chest radiographs from ASD patients.

**Conclusion:**

The ResNets model is feasible for identifying ASD through children’s chest radiographs. ResNet-10t stands out as the preferable estimation model, providing exceptional performance and clear interpretability.

## Introduction

Atrial septal defect (ASD) is one of the most common of left-to-right congenital heart disease (CHD), accounting for about 10 percent of all CHD at birth [1]. The level of hemodynamic changes in children with ASD is not uniform. Certain children have the subtle clinical signs and the difficulty of detecting heart murmurs. ASD is the most prevalent CHD in adults [2]. The left-to-right shunting of ASD raises volume load of the right heart and pulmonary circulation, which ultimately affects the cardiac morphology, resulting in an enlargement of the right atrium, right ventricle, and pulmonary artery dilation. In children with ASD, digital radiography (DR) images of the chest will illustrate a range of enlargement of the right heart silhouette, pulmonary artery dilation, and heightened pulmonary hilar shadow [3].

In recent years, increased number of children have visited doctors complaining of chest symptoms such as chest tightness, and DR examination of the chest is preferred [4]. Numerous instances of evaluations for ASD have been conducted on these children, largely as a result of pediatricians’ increasing familiarity with the form of heart shadows on chest DR image of ASD. Based on the abnormal DR and characteristic manifestations, further echocardiography are carried out to confirm ASD diagnosis. In certain cases of ASD, diagnosis may be delayed due to unremarkable chest radiographs, lack of expertise among the initial physician and/or the economic constraints of the family, which preclude the use of echocardiography [5].

With the increasing maturity of the technical conditions for artificial intelligence, deep learning has been a key technology for image recognition in the medical field. Deep learning can be used to extract the features of the target datasets, which is especially useful for identifying and analyzing objects with complicated or even unidentified characteristics [6]. Based on residual networks (ResNets), the study used a variety of learning models to develop DR images recognition for ASD. By comparing performance of detection of ASD in chest DR images and visual the extracted feature with heat maps, the value of the ResNets for the detection of ASD in DR images of chest was discussed in a preliminary manner.

## Materials and methods

### Study patients

In this study, a series of retrospective data of chest DR images of children from Women and Children’s Hospital, Qingdao University were consecutively collected from June 2017 to May 2022. Eligible chest DR images were taken from children who had undergone echocardiography and mark the images based on the echocardiogram report. According to the American Society of Echocardiography recommendations [7], echocardiography-indicated ASD defects of more than 3 mm are to be identified as ASD positive.

Inclusive criteria: All ASD-positive patients aged 1–18 years old from both inpatient and outpatient settings, healthy children from the same age range were also purposely gathered. Posteroanterior chest radiographs acquired in the standing or supine position. For each child, we only included one chest radiograph and excluded repeated chest radiographs or those taken as part of a follow-up. Exclusion criteria: Fuzzy image quality, posture irregularities, other types of CHD or pulmonary vascular disease, chest space occupation or foreign body, lung infection or trauma, post cardiac, or chest surgical procedure.

The ethics commission of our establishment examined and accepted the protocol of the study. The need for an informed consent has been waived, since the images were acquired during clinical practice by patients who have given their consent to the use of the data for research purposes. Patients are guaranteed the right to opt out of the study.

### Examination and image acquisition

Chest radiographs were obtained by using a DRX-Evolution Plus (Reichert, USA), with fully automatic exposure. All chest radiographs are extracted in Digital Imaging and Communications in Medicine (DICOM) format, then personal information including name, gender, and age are taken out of the images. Since DICOM files have different window widths, window levels and density values, all images were converted into JPG format via image pre-processing to ensure consistency. To begin with, the chest radiographs were adjusted to 320 × 320-pixel JPG files. The longer side was reduced to 320 pixels while retaining the aspect ratio. To complete the size, the shorter side of the radiographs was filled with black until it reached 320 pixels. To further enhance the performance of the model, the training images were augmented via random transformations, such as flip, flop, and rotation, due to the symmetrical nature of the lungs.

Every chest radiograph is reviewed independently by doctors A and B (both with 3-year qualification) without disagreement. The size of the image, window width, and window position can be adjusted in the process of reviewing the radiographs, and time is not restricted. Echocardiography to evaluate ASD was performed by using an iE33 (Philips Medical Systems), the same child was independently performed by two ultrasound physicians with qualifications of more than 3 years, and the results of echocardiography were confirmed to be consistent.

### ResNets model development

The study relies on a deep learning algorithm based on the ResNets, a modification from the traditional Convolutional Neural Network (CNN) [8]. Besides the standard convolutional layer output, ResNets have a section that connects the input directly to the output, which is added mathematically to the convolutional output to create the final output.

In this study, six models (ResNet-10t, ResNet-18D, ResNet-14t, RexNet-200, ResNet-18, and ResNet-26D) were chosen for training and learning, and the validation dataset was used for modification and enhancement, with the model performance assessed on the test dataset.

The study was conducted in a 12-core Intel (R) Xeon (R) Platinum 8255C CPU and GeForce RTX 3090 GPU environment, with PyTorch as the chosen framework and a random seed of 1024. Python is employed as the programming language in this investigation, with the upscaling process realized through the transformation feature of PyTorch. During the ResNet model training process, a weighted random sampling approach and a weighted cross-entropy loss function were used to address the effect of data imbalance on performance degradation.

### Statistical analysis

The aggregate of the gathered chest radiography images of ASD patients was split into training, validation, and test dataset according to an 8: 1: 1 ratio. Patient-level allocation makes sure that there is no duplication of images or participants between the distinct datasets. The evaluation methods used include accuracy, recall (*R*), precision (*P*), and F1-score (F_1_). They are calculated as follows:

$${\text{Accuracy}} = N_{{{\text{tp}}}} + N_{{{\text{tn}}}} /\left( {N_{{{\text{tp}}}} + N_{{{\text{tn}}}} + N_{{{\text{fp}}}} + N_{{{\text{fn}}}} } \right)$$

$$R = N_{{{\text{tp}}}} /\left( {N_{{{\text{tp}}}} + N_{{{\text{fn}}}} } \right)$$

$$P = N_{{{\text{tp}}}} /\left( {N_{{{\text{tp}}}} + N_{{{\text{fp}}}} } \right)$$

$$F_{1} = 2PR/(R + P)$$

where tp is the number of true-positive samples, tn is the true-negative samples, fp is the false-positive samples, and fn is the false-negative samples.

### Visualizing regions of interest for the trained model by using heat maps

Two ResNets models with better performance were chosen through comparison, and heat maps were constructed for each chest DR image to illustrate the concentration of the deep learning model. A classification activation graph was utilized to create a visual representation of chest radiographs, and the source code is accessible on the internet [9, 10]. The heat map of classification is used to visualize the area of interest in the DR image of the chest. The Gradient-weighted Class Activation Mapping (Grad-CAM) technique highlights the regions of an input image that the model is more focused on when classifying, signifying that the feature maps produced in the final convolutional layer provide spatial information which assists in recognizing visual features and differentiating the designated categories more precisely. After applying the visual model to test dataset, two cardiologists from the pediatric cardiology field with 8 years of clinical experience need to assess the anatomic regions demonstrated on the heat map of the chest image from the same ASD patient.

**Fig. 1:**
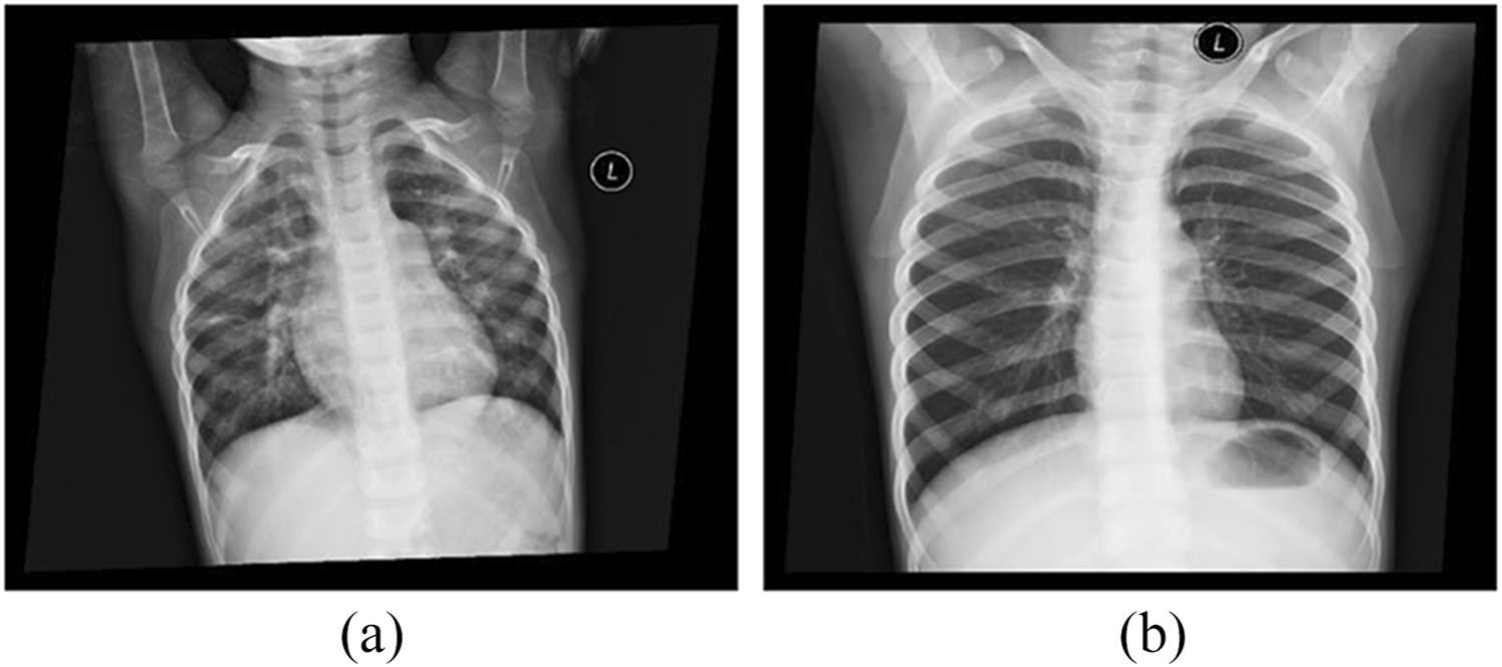
The the chest DR images of children. **a** The images of atrial septal defect child. **b** The images of health child

Since the chest DR images of ASD include enlargement of the right heart, peculation of the pulmonary artery, and hilar shadow enhancement (Fig. 1a), the position of the heat map distribution in these areas was visually evaluated. When there is disagreement among doctors, consensus is reached through discussion.

## Results

### Datasets

A total of 2105 images of children with ASD were included in the study (mean age 4.14 ± 2.73 years, 54% male). The data were divided into 1685 training dataset, 210 verification dataset and 210 test dataset by Python random function method according to 8:1:1. Three datasets were assigned an corresponding amount of healthy children’s chest DR images in a 1:1 ratio. The details of the three datasets are shown in Table 1.

**Table 1:** Dataset information

Dataset	Train and validation		Test	
	ASD	Health	ASD	Health
Number of cases	*n* = 1895	*n* = 1900	*n* = 210	*n* = 200
Age (years)	4.62 ± 1.92	5.31 ± 1.16	3.91 ± 2.15	4.22 ± 1.57
Gender (male/female)	1035/860	988/912	102/108	98/102
Cardiothoracic ratio	0.52 ± 0.07	0.48 ± 0.10	0.53 ± 011	0.47 ± 0.08
Atrial septal defect (mm)	7.32 ± 3.16	–	8.45 ± 2.05	–

### Model evaluation

The test dataset in the study demonstrated that ResNet-10t and ResNet-18D had the highest precision, accuracy, recall and F1-score for predicting ASD from chest DR images, as seen in Table 2.

**Table 2:** Test dataset model results

	Precision	Accuracy	Recall	F1-score
ResNet-10t	0.92	0.91	0.90	0.91
ResNet-18D	0.93	0.91	0.90	0.91
ResNet-14t	0.88	0.88	0.88	0.88
RexNet-200	0.78	0.86	0.86	0.82
ResNet-18	0.85	0.84	0.84	0.84
ResNet-26D	0.86	0.78	0.78	0.82

**Fig.2:**
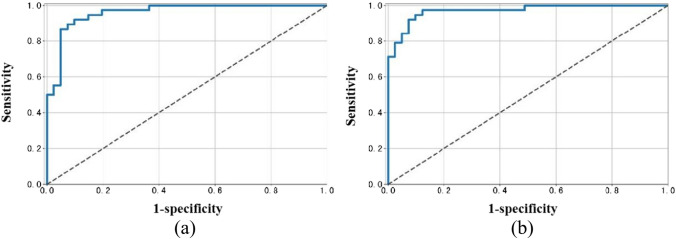
The area under the curve of the model using the test dataset. **a** The ResNet-18D. **b** The ResNet-10t

As shown in Fig. 2, the area under the curve for ResNet-10t and ResNet-18D when applied to detection of ASD in chest radiographs was 0.97 (95% CI 0.93, 1.00) and 0.96 (95% CI 0.91, 1.00), respectively.

**Fig.3:**
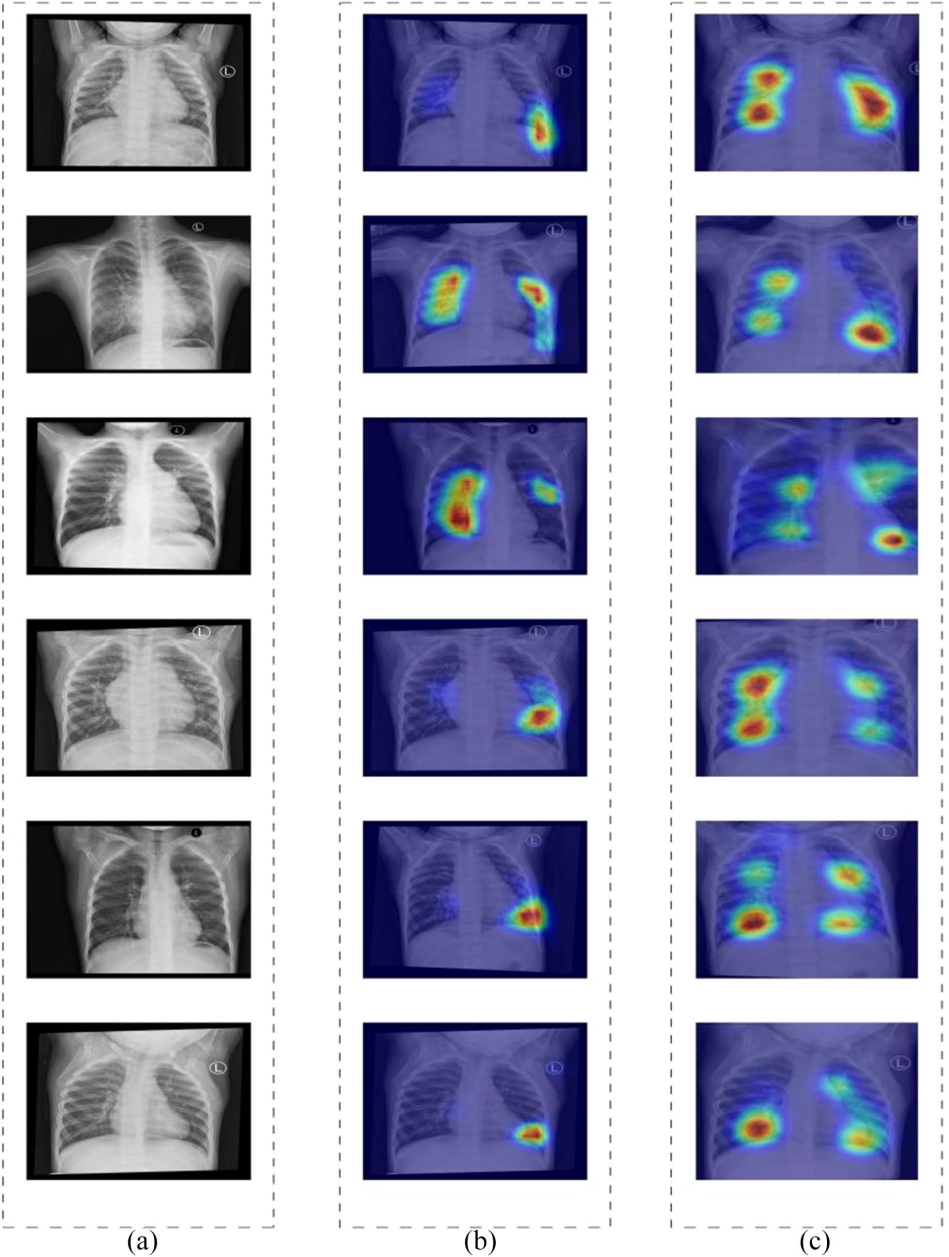
Representative heat maps of chest radiographs showing the anatomical interest region consistent with the ASD. **a** is digital radiography images of the chest from atrial septal defect patients. **b** The ResNet-18D heat maps. **c** The ResNet-10t heat maps

Analysis of the DR images from ASD children in test dataset revealed that the ResNet-10t was most prominently distributed in the right atrium (98.5%, 190/192) and pulmonary artery segment (97.9%, 188/192) in the majority of images. The heat map of the ResNet-18D was not focused on the area of interest, with the heat maps of the right atrium and pulmonary artery segment registering at 53% and 78%, respectively. The heat map effects of the two models are shown in Fig. 3.

## Discussion

The appearance or the severity of the clinical symptoms of children with ASD is primarily affected by the size of defect. In infancy, the thickness of left and right ventricular wall is similar, with a minimal difference in pressure, meaning that the left-to-right shunt is not very pronounced and generally does not produce any noticeable clinical signs. With age, the compliance of the right ventricle wall increases, the pressure of the systemic circulation increases, and the horizontal shunt increases, resulting in pulmonary congestion and reduced amount of blood in the systemic circulation. Pneumonia is a common affliction of children with ASD, causing retardation in their development. As family living conditions improve, parents may not take into account the children’s slow weight gain and excessive sweating. Finding these signals is challenging, most people with ASD can still lead a normal life when they reach adulthood.

In recent years, there has been an upsurge in the amount of children in outpatients clinics experiencing chest tightness, sighing, chest pain and other primary indicators, and chest DR has become a typical initial diagnostic tool in children, which is straightforward and economically sensible. This examination provides significant insight into pediatric chest pathology. Nonetheless, analyzing children’s chest X-ray images can be challenging. In addition to lung imaging, physicians are more attuned to the shape of the heart shadow with more recognizing experience [11]. Echocardiography has often been used to screen for ASD from abnormal performers. For many children with ASD, there are no visible changes in the hemodynamic system, and chest radiographs usually appear normal. However, other physicians who are not cardiovascular experts or those with lack experience may have difficulty identifying any abnormal cardiac changes on chest radiographs. In addition, echocardiography is expensive, as routine screening for chest abnormalities is difficult to accept by ordinary families in developing countries, resulting in delay in diagnosis for children with ASD. Machine learning, especially deep learning, presents a great opportunity to improve medical imaging, expediting diagnostics and normalizing interpretation. Based on what we know now, this is the first time deep learning has been demonstrated that could effectively assist in the early detection of ASD in children through chest radiographs in our study.

Utilizing CNN is a common approach of constructing a deep learning system. CNN can accumulate data from each component of the picture and have potent feature learning aptitude. ResNet has become a well-known deep learning model for image classification because of its capacity to overcome the vanishing gradient problem, which is often encountered during training of traditional convolutional neural networks (CNN) using residual mapping. ResNets is utilized to draw out image features sans interference from shape, rib and other related noise. The ResNet mechanism has also been incorporated into more machine learning models, greatly improving the model ability to identify different types of lung diseases with chest X-rays.

In the past 5 years, there has been a rapid expansion in the application of artificial intelligence technology in examining chest X-ray images, the majority of which have been concentrated on adults, and its application in pediatric is still scant [12]. It is common knowledge that the imaging manifestations of children’s chest will alter throughout their normal growth and development. Physiological modifications in chest wall, lung, ossification center, and thymus width in children. Pathological issues of distinctive deformities such as congenital pulmonary cystadenoma. The above issues present additional challenges in the development of artificial intelligence models based on chest X-ray images for identifying lung diseases in children [13].

Luo et al. [14] showed that a new model based on YOLOv5 and ResNet-50 to identify abnormal chest radiographs, mean average precision (mAP) was 0.010, 0.020 and 0.023 higher than the mAP values of YOLOv5, Fast RCNN and EfficientDet models, respectively. And, the precision of the new model were 0.512, 0.018, 0.027 and 0.033, higher than YOLOv5, Fast RCNN and EfficientDet. The classification accuracy of the training model ResNet-50 based on CNN for lung cancer, pneumothorax, tuberculosis, pneumonia and other chest diseases was 96.15%, and that of Vgg-19 and Inception V3 were 95.61% and 95.16%, respectively [15].

Because the heart is positioned and shaped in a relatively stable manner within the chest cavity, it is less affected by the development of the lungs and bones. Thus, future research on the use of artificial intelligence technology for the diagnosis of chest X-ray images will concentrate primarily on the cardiovascular diseases [16]. Through the use of deep learning, research into cardiovascular diseases has been done by evaluating chest X-rays, such as automatic detection of cardiomegaly, valvular disease and cardiac function, classification of pulmonary hypertension and atrial fibrillation and prediction of pulmonary to systemic flow ratio in patients with congenital heart disease [6, 17–20]. The findings of this research point to the potential of deep learning-based methods to objectively and quantitatively evaluate cardiac morphology and pulmonary circulation blood volume as seen on chest X-rays, allowing for the distinction or diagnosis of heart conditions. Ueda et al. [21] first explored research on create and validate ResNet-50 for mitral regurgitation through chest radiographs recognition. The area under the curve, sensitivity, specificity, accuracy, positive predictive value and negative predictive value of the artificial intelligence model were 0.80 (95% CI: 0.77, 0.82), 71% (95% CI 67, 75), 74% (95% CI 70, 77), 73% (95% CI 70, 75), 68% (95% CI 64, 72) and 77% (95% CI: 73, 80), and the model has the potential to distinguish between patients with and without mitral regurgitation by means of the areas of the left atrium, left ventricle, and superior vena cava in chest radiographs [21]. Lee et al. [8] used ResNet-18 model to identify chest radiographs to diagnose acute thoracic aortic dissection. The accuracy was 90.20%, the precision was 75.00%, the recall was 94.44% and the F1-score was 83.61%, the model offers excellent performance and is able to accurately detect acute thoracic aortic dissection based on plain chest radiography.

In children with ASD, due to increased left-to-right shunt, the right cardiac preload increases, leading to enlargement of the right atrium and right ventricle, increased pulmonary circulation blood volume and pulmonary artery dilation. Chest radiographs demonstrate a more prominent cardiac shadow, which can be observed as a higher cardiothoracic ratio. It is generally observed that the right side of the heart is more saturated in children with ASD. In the eventuality of pulmonary congestion, the pulmonary artery section is conspicuously projecting, the hilar shadow is dilated, the lung texture is bloated and the vascularity is particularly pronounced in the lower right lung [2]. Consequently, with the modifications in the characteristics of ASD chest radiographs, our study is bringing in artificial intelligence technology to upgrade the recognition accuracy of chest radiographs and serve as a premise for echocardiography, thus enabling an accurate diagnosis of ASD as quickly as possible without raising the financial cost for the family. With the comparison of a variety of models, this study revealed that the recognition accuracy of the ResNet-10t was exceptionally high, reaching 92% after training, notably better than other models. In this study, the Grad-CAM visual perception hotspot analysis of the ResNet-10t is carried out, and it was found that the local features capturing the model focused on the cardiac margin right and the pulmonary arterial segment of the heart shadow, which further confirms the high credibility of the model performance.

### Limitations

All participants were over 1 year of age, mainly because of the substantial heart shadow in the first year of life and the cardiothoracic ratio of 0.55 in the chest radiograph diagnostic criteria. Children diagnosed with ASD in this age group tend to have large defects, typical clinical manifestations and imaging manifestations, potentially leading to an erroneous boost in the accuracy of the learning model. Thus, this study shall encompass this age group in the foreseeable future, and it is necessary to adjust the learning model algorithm or develop a new learning model directed to differentiate. The thymus is a significant lymphatic organ located in the front middle part of the chest cavity that grows throughout childhood and peaks in adolescence. Consequently, the thymus shadow in the chest radiographs is a soft tissue density with a smooth border, making it hard to separate from the heart shadow. This investigation is uncertain of the composition of thymus interference factors in image recognition, improving the accuracy of recognition is a main focus of the following research.

In conclusion, the ResNets model is feasible for identifying ASD through children’s chest radiographs. The ResNet-10t in this study was more effective than the main detection models when used on chest DR images of ASD. In subsequent studies, transformer models will be designed based on this model to expedite the identification and examination of chest DR images of ASD across all ages, which has great clinical and societal value.
